# Use of Postpartum Birth Control in Rural Women in Southwest Guatemala: Analysis of a Quality-Improvement Database

**DOI:** 10.26502/ogr069

**Published:** 2021-10-23

**Authors:** Kathryn Feller, Claudia Rivera, Amy S Nacht, Saskia Bunge-Montes, Andrea Jimenez-Zambrano, Molly Lamb, Gretchen Heinrichs, Antonio Bolanos, Edwin Asturias, Sephen Berman, Margo S Harrison

**Affiliations:** 1 University of Colorado Anschutz Medical Campus, Colorado, USA; 2 Fundación para la Salud Integral de los Guatemaltecos, Retalhuleu, Guatemala; 3 Denver Health, Denver, Colorado, USA

**Keywords:** Postpartum Birth Control, Guatemala

## Abstract

**Objective:**

Our objective was to observe the prevalence of postpartum contraceptive use in a population of rural women in Southwest Guatemala by type, and to determine characteristics associated with long-acting reversible contraceptive (LARC) use and sterilization.

**Methods:**

We conducted a secondary analysis of prospectively collected quality improvement data from a cohort of postpartum women. We compared women intending to use or already using contraception to those not intending to utilize a method; bivariate comparisons were used to determine if there were differences in characteristics between these groups. If differences occurred (p < 0.2), those covariates were included in multivariable regression analyses to determine characteristics associated with use, and then specifically with LARC use and sterilization.

**Results:**

In a cohort of 424 women who were surveyed between 2015–2017, the average age was 23 years old, and the prevalence of use or plan to use postpartum contraception was 87.5%. Women with a parity of 2 – 3 were 10% more likely to use any form of postpartum birth control (RR 1.1, CI [1.01, 1.2]) compared to primiparous women. Women who were married were also more likely to use a postpartum method (RR > 10, CI [>10,>10]). The prevalence of LARC use was low (4.0%), and women were more likely to choose this method if they were employed (RR 3.5 CI [1.1, 11.3]).

Regarding sterilization, women with a parity of greater than one compared to primiparous women had an increased likelihood of sterilization (RR 3.6 CI [2.5,4.9]); each year a woman aged was associated with a 10% increased likelihood of postpartum sterilization (RR 1.1 CI [1.01,1.08]). Women were also more likely to choose sterilization if delivered by a skilled birth attendant (RR 1.8 CI [1.1,2.9]) or by cesarean birth (RR 2.1 CI [1.4,3.1]).

**Conclusion:**

In this cohort, married women of higher parity were more likely to use postpartum contraception, with employed women more likely to use a LARC method. Older women of higher parity who were delivered by a skilled attendant by cesarean birth were the most likely to pursue sterilization.

## 1. Introduction

Postpartum contraception is important to properly space and prevent undesired and unintended pregnancies [1]. Effective postpartum contraception can aid in prevention of adverse health outcomes such as: maternal and infant mortality, financial hardship, domestic violence, unsafe abortions, and obstetrical complications [2]. Worldwide, of the 1.9 billion women of reproductive age, 57% have an unmet need for family planning [3]. Of that 57%, 17% of women who want to avoid pregnancy do not use any contraceptive method [3]. Postpartum contraception can avert more than 30% of maternal deaths and 10% of child mortality if pregnancies are spaced greater than 2 years apart [4]. Regarding global contraceptive method use, 45.2% of users rely on long-acting or permanent methods of contraception, including male & female sterilization, and intrauterine devices (IUD), while 46.1% rely on a short-acting methods including condoms, the contraceptive pill, and injectables [5]. The remaining 8.7% rely on traditional methods, including withdrawal and rhythm methods [5].

The method of contraception varies widely by world region [5]. In Latin American and the Caribbean, modern contraceptive use is 58%, with 6.1% using long-acting reversible contraceptives (LARC) and 16% using female sterilization [5]. However, the unmet need for family planning services is still estimated to be about 10.7% in this region [5]. It is also estimated that 66% of all reproductive aged women age 15–49 in Latin America wish to avoid pregnancy [5].

In Guatemala, according to the Demographic and Health Survey from 2014–2015, there was a 74.5% demand for family planning among reproductive age women accompanied by a 13.9% unmet need for modern contraception [6]. Overall contraceptive use rates in Guatemala are 41.5% nationally, but only 27.5% in low-resource areas [6]. The low use of LARC methods has been attributed to lack of knowledge of modern contraceptive methods [6]. This analysis observes prevalence of postpartum contraceptive use in a population of rural women in Southwest Guatemala. We hypothesized that most women would use postpartum contraception but would be less likely to use LARC as this was not conveniently available in the community.

Our primary outcome was the use of postpartum contraception by type, with characteristics associated with LARC use and sterilization as secondary outcomes.

## 2. Methods

### 2.1 Setting

The University of Colorado, in partnership with a local agribusiness, founded a health service organization in the Southwest Guatemalan lowlands to provide healthcare to migrant workers who experience poverty and poor pregnancy outcomes [7]. The organization supports community-based maternal and child health programming [7]. Quality improvement data on antepartum, intrapartum, and postpartum care of mother and baby is collected over the course of pregnancy by community health nurses [7]. Women are visited over the course of pregnancy and postpartum by the nurses in their homes, and their children are followed for pediatric care through two years of age [7].

### 2.2 Population

This study analyzed the prospectively collected quality improvement data on the question of postpartum contraceptive use among a convenience sample of women enrolled in the program who were seen for their postpartum visit between June 1, 2015, and July 1, 2017. Women are seen for their final maternal visit in the community-program in their homes around 40 days postpartum. At this time, in addition to other topics, they are asked about postpartum contraceptive use. The data is collected by the community health nurses on tablets in the REDCap application and transmitted securely to password-protected servers at the University of Colorado. Individual consent is not obtained, but the quality improvement database has ethics approval from the Colorado Multiple Institutional Review Board (COMIRB #150909).

### 2.3 Outcomes

This secondary analysis of the quality improvement database has the primary outcome of postpartum contraceptive use. We wished to observe the prevalence and type of contraceptives used among our convenience sample of postpartum mothers in the community. Secondarily, we wanted to identify characteristics or subpopulations of women using LARC and sterilization as their initial method of choice.

### 2.4 Analysis

A descriptive analysis was performed to determine prevalence of postpartum contraceptive use as well as to summarize the demographics of postpartum women by whether they were using or planning on using postpartum contraception. This question was asked one month after birth at the final postpartum visit. Bivariate comparisons of sociodemographic, antepartum, and intrapartum characteristics were made using Pearson’s chi-squared test for nominal categorical variables, unless there was a low cell size in which case Fisher’s exact test was used. The Kruskal-Wallis test was used for comparison of continuous variables. Variables with a p < 0.20 were considered statistically significant. Multivariable generalized regressions were applied to find the risk and odds first of using or planning on using postpartum contraception, then of using LARC, and finally of sterilization. The data were analyzed using STATA software version 15.2 (StataCorp LP, College Station, TX, USA).

## 3. Results

**Figure 1:**
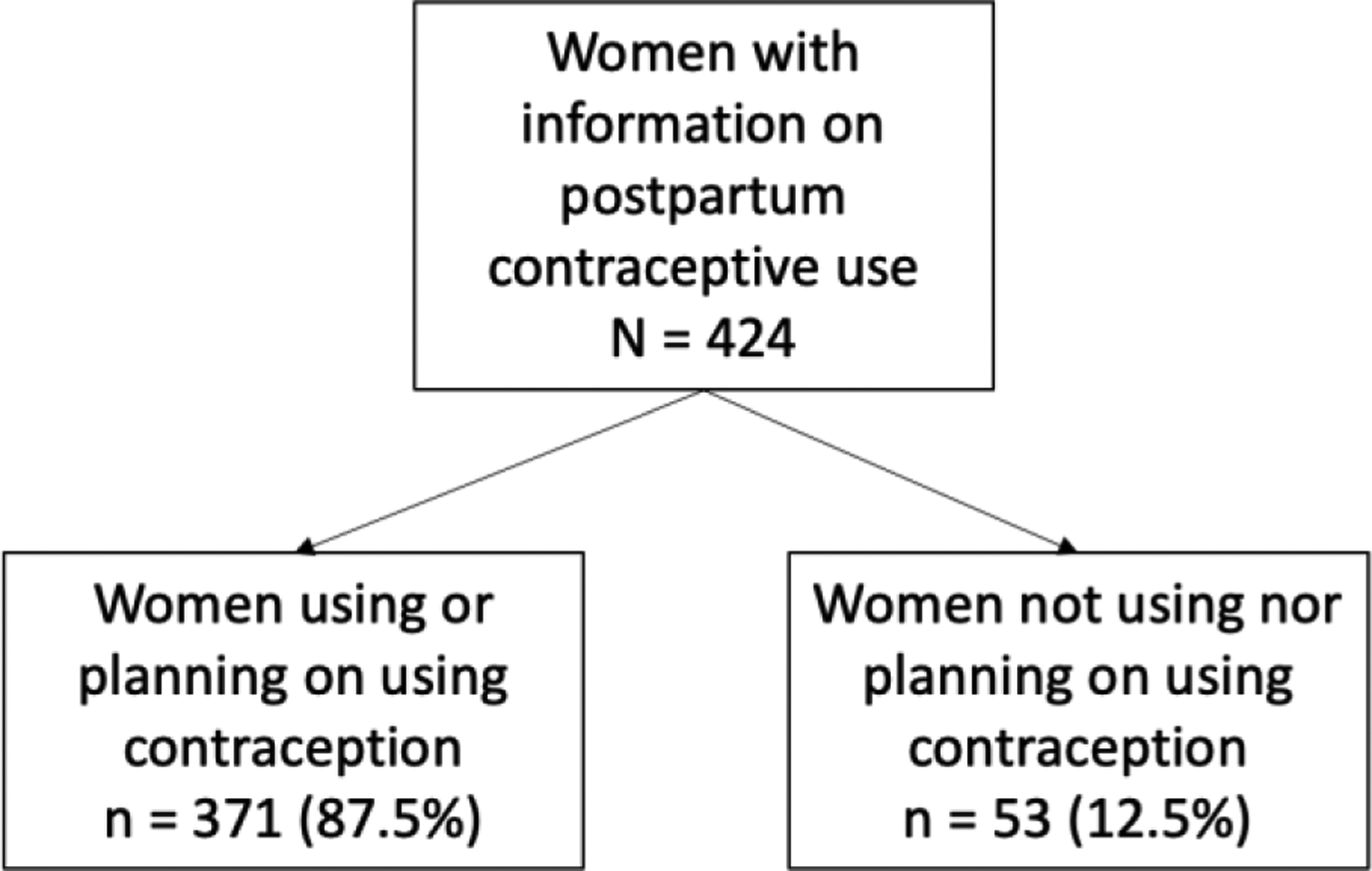
Consort Diagram.

As shown in Figure 1, of the 424 women who were surveyed in our study between 2015–2017, 371 women (87.5%) were using or planning to use contraception, and 53 women (12.5%) were not using or not planning on using any form of contraception when they were asked at their one month postpartum visit. In our study population, as shown in Table 1, most women (47.5%, n = 193) were multiparous, were married or living with a partner (99.5%, n = 394), had an elementary school education (45.6%, n = 193), and were not employed (93.4%, n = 395). The majority of women (62.2%, n = 263) had not been trying or planning on becoming pregnant at the time of conception. The mean age of the population was 22.8 years (SD = 0.3). As shown in the last column of Table 1, in bivariate analyses comparing uptake of postpartum contraception, women with higher parity of 2–3 children (49.9%, n = 178, p-value <0.001) were more likely to use postpartum contraception than primiparous women. Women who used postpartum contraception were more likely to be married/living with partner (88.8%, n = 394 vs 2, p < 0.01), and were more likely to be older (23.1 years vs 20.2, p-value <0.001). Level of education, working for the local agriculture company, and pregnancy intention were not statistically different between the comparator groups.

**Figure 2:**
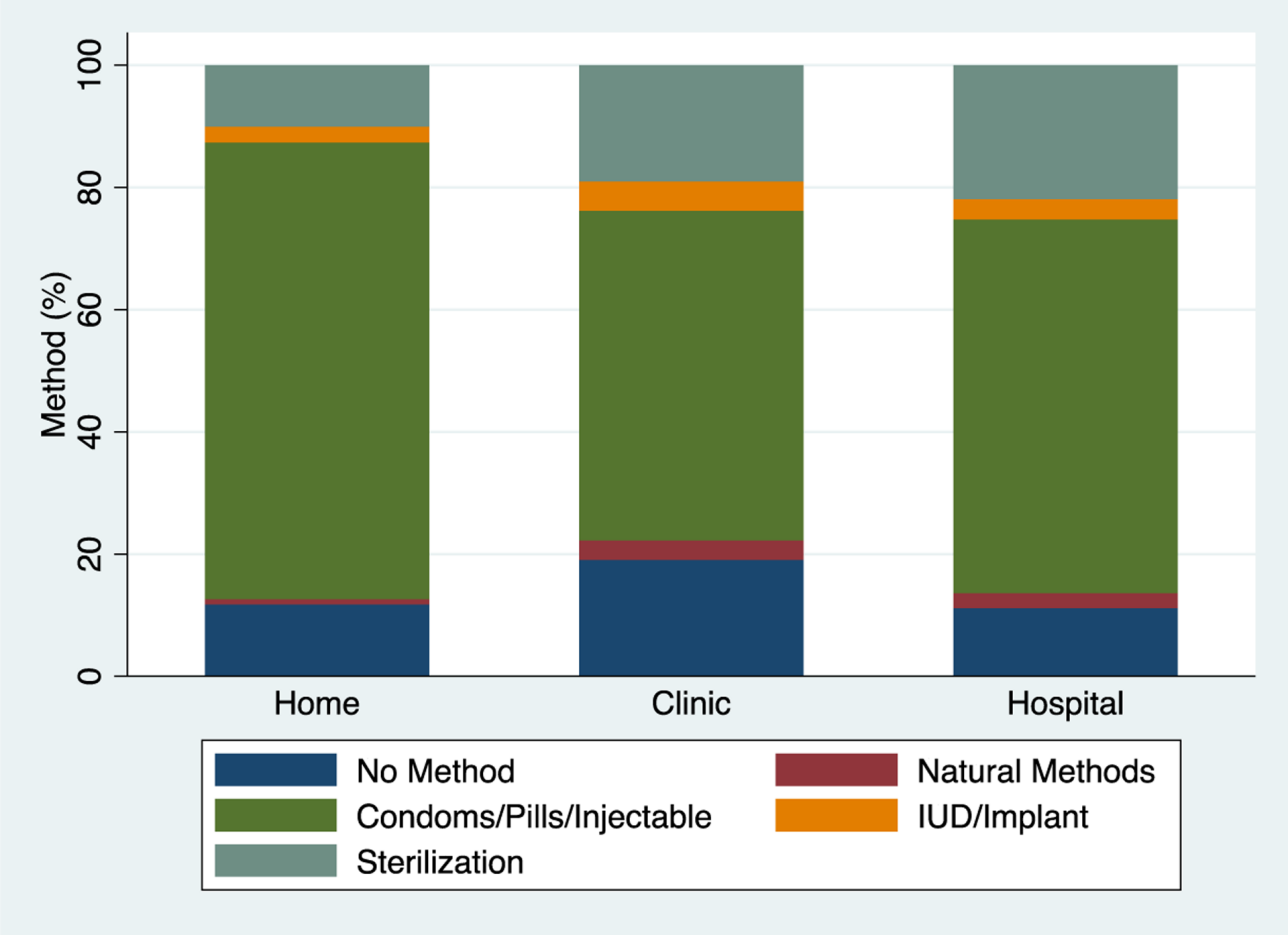
Method of Postpartum Contraception Used Among Utilizers by Delivery Location, 2015 – 2017.

Figure 2 illustrates method of postpartum contraceptive use among women who initiated a method, by delivery location. The figure shows that women who delivered in the home more frequently used short acting methods of contraception (condoms/contraceptive pills/injectables) as compared to women who delivered in a facility setting (clinic or hospital). Women who delivered in a facility setting more frequently used long-acting methods of contraception (IUD/implant) or permanent sterilization than women who delivered at home. In the bivariate analysis of antepartum care and delivery planning by initiation of postpartum contraception that is shown in Table 2, most of the population planned to be delivered by a physician (74.7%, n = 316), had less than 4 prenatal visits (53.8%, n = 228), and planned to give birth at a facility (76.9%, n = 326). There were no statistically significant differences between the women who used postpartum contraception and those that did not by these characteristics. Table 3 shows bivariate analyses of the delivery characteristics by the comparator groups with most of the population delivering at greater than 37 weeks gestational age (89.1%, n = 378), with a physician (69.6%, n=295), by vaginal birth (59.25%, n=251), and at a hospital (57.1%, n=242). No delivery characteristics were found to be statistically different between the groups. A Spearman’s correlation was used to assess the relationship between physician birth attendant and cesarean birth using 430 observations; there was a positive correlation of rho = 0.55, p < 0.001.

In our first multivariable analysis (Table 4) of demographic, antepartum and delivery characteristics associated with any postpartum birth control use, women with a parity of 2 – 3 were 10% more likely to use any form of postpartum birth control (RR 1.1, CI [1.01, 1.2]) compared to primiparous women. Women who were married were also statistically more likely to use a postpartum method (RR > 10, CI [>10,>10]). Table 5 is a second multivariable model that looks at characteristics associated with postpartum LARC use. Results significant in bivariate comparisons of women who chose LARC compared to those who chose another method (data not shown), were included in the model. These included employment status and parity; only employment status increased the likelihood of using postpartum LARC significantly (RR 3.5 CI [1.1, 11.3]). Table 6 observes characteristics associated with postpartum sterilization. Women with a parity of greater than one compared to primiparous women had an increased likelihood of sterilization (RR 3.6 CI [2.5,4.9]). Each year a woman aged (average age of the population was 23 years old) was associated with a 10% increased likelihood of postpartum sterilization (RR 1.1 CI [1.01,1.08]). Women were also almost 2 times as likely to choose postpartum sterilization if delivered by a skilled birth attendant than an unskilled attendant (RR 1.8 CI [1.1,2.9]) and 2.1 times as likely to choose postpartum sterilization if delivered via cesarean birth (RR 2.1 CI [1.4,3.1]).

## 4. Discussion

This historical analysis of use of postpartum contraception in this cohort of rural Guatemalan women found that married women of higher parity were more likely to use postpartum contraception overall, with employed women more likely to use a LARC method, and with older, more parous women delivered by a skilled attendant and by cesarean being the most likely population to choose sterilization. Overall rates of postpartum contraceptive use or intended use were high at 87.5%. Women delivering at home were more likely to pursue short-acting methods while women delivering at the hospital were more likely to pursue sterilization. It is not a novel finding that women who were older, more parous, and married were more likely to use postpartum contraception. This result supports what has been found in other global settings as well as in Latin America and lends external validity to our findings [8]. This finding suggests that younger, less parous women who are uncoupleed may be at risk for an unmet need for contraception [8]. It is interesting to note that the difference between “young” and “old” in this context is only three years, so there is a tight window for intervention that should focus on older adolescents for quality improvement interventions. It was an unexpected finding (illustrated in Figure 2) that women who delivered in private clinics had the highest prevalence of not using or not planning on using a postpartum contraceptive method. Prior research in Guatemala has shown that service delivery factors are associated with uptake of postpartum contraception, and that facilities with strong community ties have increased uptake [9]. The longevity of the maternal healthcare program in this region and the strong relationship with the community likely accounts for high uptake of postpartum contraception in this cohort generally, but may also explain this finding regarding clinic birth. Women in this region who deliver in the clinic setting usually do so at private clinics that are not affiliated with our antepartum and postpartum care program, which suggests there is an opportunity to improve relationships with local clinics to support their administration of postpartum contraceptives.

Given the finding that the difference between “older” women pursuing sterilization and “young” women using short-acting methods, this analysis highlights the need for increased LARC use in this community as an alternative to sterilization. Sterilization among young women has been associated with regret [10]. The overall prevalence of LARC use was lower than expected (4.0%); we hypothesize this is due to the fact that LARC devices are expensive and not readily available in this region [11]. Prior research in Guatemala has found that when devices are provided free of charge in communities with limited access to health services, overall uptake of LARC was 79.2% and more than half of women switched to LARC from short-acting hormonal methods [12]. That study also highlighted that low utilization of LARC may be related to service delivery factors [12]. Our results support these findings that postpartum contraception and LARC, specifically, if made readily accessible to geographically isolated and underserved communities in Guatemala, will likely be utilized. We hypothesize that there would be, accordingly, less of a dependence on sterilization, which is more likely in “older” women of higher parity who deliver by cesarean birth. Our preliminary qualitative data from the referral hospital in this region has suggested that at the third cesarean birth women are often sterilized, which is a non-evidence based recommendation that is practiced regularly at the facility (data under review). Strengths of the analysis are the relatively large sample size, the ability to report on postpartum use in an otherwise geographically isolated and underserved community, and the identification of gaps in care that will support future quality improvement and research activities. This study is limited primarily by the question that was asked, which was, “are you using or planning on using postpartum contraception?”. We intend, based on these results, to separate the two questions to get a more detailed understanding of actual versus planned method use. Women in this region have a tradition of avoiding sexual intercourse for 40 days postpartum, which is important to consider when collecting postpartum contraceptive uptake data [13]. Additionally, the data was collected by self-report in a convenience sample of women.

In conclusion, sensitization about and intention to use postpartum contraception is high in this rural Guatemalan community, likely due to the excellent contraceptive education provided through regional maternal health services. However, uptake of LARC methods is low, which parallels regional findings [14, 15]. This analysis highlights the need for a prospective study that considers service and financial factors that may be barriers to access, and the importance of focusing on adolescent and young adult populations.

## Figures and Tables

**Table 1:** Demographic Characteristics of Women by Initiation of Postpartum Contraception, 2015 – 2017.

Characteristics	Total	No	Yes	p-value
**Age**	**n = 423**	**n = 53**	**n = 370**	<0.001^c^
Mean (Standard Deviation)	22.8 (0.3)	20.2 (0.6)	23.1 (0.3)	
**Parity**	**n = 423**	**n = 53**	**n = 371**	<0.001^b^
1	134 (33.0%)	30 (61.2%)	104 (29.1%)	
2 – 3	193 (47.5%)	15 (30.6)	178 (49.9)	
4+	79 (19.5%)	4 (8.2)	75 (21.0)	
**Marital Status**	**n = 391**	**n = 41**	**n = 350**	0.01^b^
Single/ Divorced/Separated/ Widowed	2 (0.5%)	2 (4.9%)	0 (0%)	
Married/Living with a Partner	394 (99.5%)	39 (95.1%)	350 (100%)	
**Education**	**n = 423**	**n = 53**	**n = 370**	0.1^a^
No formal education	146 (34.5%)	13 (24.5%)	133 (36.0%)	
Elementary school (Between 1 – 6)	193 (45.6%)	25 (47.2%)	168 (45.4%)	
High school/Technical School	84 (19.9%)	15 (28.3%)	69 (18.7%)	
**Do you work for the local agricultural company?**	**n = 423**	**n = 53**	**n = 370**	0.4^a^
Yes	28 (6.6%)	5 (9.4%)	23 (6.2%)	
**Were you trying or wanting to become pregnant when you became pregnant with this baby?**	**n = 423**	**n = 52**	**n = 371**	0.6^a^
Yes	160 (37.8%)	18 (34.6)	142 (38.3)	

a chi^2^ test;

b fisher’s exact test;

c t-test

**Table 2:** Antepartum Care and Delivery Planning by Initiation of Postpartum Contraception, 2015 – 2017.

	Total	No	Yes	p-value
**Who will attend the birth?**	**n = 424**	**n = 53**	**n = 370**	0.06^a^
I don’t know	15 (3.6%)	1 (1.9%)	14 (3.8%)	
Midwife/Nurse/Community health worker	92 (21.8%)	18 (34.0%)	74 (20.0%)	
Doctor	316 (74.7%)	34 (64.2%)	282 (76.2%)	
**How many prenatal visits did you receive during this pregnancy?**	**n = 424**	**n = 53**	**n = 371**	0.3^a^
< 4	228 (53.8%)	32 (60.4%)	196 (52.8%)	
4+	196 (46.2%)	21 (39.6%)	175 (47.2%)	
**Where do you plan to give birth?**	**n = 424**	**n = 53**	**n = 371**	0.2^a^
I don’t know	19 (4.5%)	2 (3.8%)	17 (4.6%)	
Home of a Midwife/My home or home of family member	79 (18.6%)	15 (28.3%)	64 (17.3%)	
Hospital/Clinic/Birth Center	326 (76.9%)	36 (67.9%)	290 (78.2%)	

a: chi^2^ test

**Table 3:** Delivery Characteristics by Initiation of Postpartum Contraception, 2015 – 2017.

Characteristics	Total	No	Yes	p-value
**Gestational Age at Delivery**	**n = 424**	**n = 53**	**n = 371**	0.4^a^
<37 weeks	46 (10.9%)	4 (7.6%)	42 (11.3%)	
>= 37 weeks	378 (89.1%)	49 (92.5%)	329 (88.7%)	
**Delivery Attendant**	**n = 424**	**n = 53**	**n = 371**	0.4^a^
Midwife/Nurse/Community health worker	122 (28.8%)	14 (26.4%)	108 (29.1%)	
Nurse	7 (1.6%)	2 (3.8%)	5 (1.4%)	
Doctor	295 (69.6%)	37 (69.8%)	258 (69.5%)	
**Method of Delivery**	**n = 424**	**n = 53**	**n = 371**	0.9^a^
Vaginal	251 (59.2%)	32 (60.4%)	219 (59.0%)	
Cesarean	173 (40.8%)	21 (39.6%)	152 (41.0%)	
**Delivery Location**	**n = 424**	**n = 53**	**n = 371**	0.2^a^
Home of a Midwife/my home or home of a family member	119 (28.1%)	14 (26.4%)	105 (28.3%)	
Clinic/Birth Center	63 (14.9%)	12 (22.6%)	51 (13.7%)	
Hospital	242 (57.1%)	27 (50.9%)	215 (58.0%)	

a: chi^2^ test

**Table 4:** Multivariable Analyses of Patient, Antepartum, and Delivery Characteristics Associated with Postpartum Birth Control Use.

Characteristic	Relative Risk	Confidence Interval	P-Value
Parity of 2 – 3 compared to 1	1.1	1.01,1.2	0.03
Married compared to not married	>10	>10, >10	<0.001

Note: this model used a Poisson regression with robust error variance; variables different in bivariate comparisons to <0.20 were parity, education, age, desired birth attendant, desired birth location, actual birth attendant, and actual birth location. Only significant outcomes are presented in above table.

**Table 5:** Multivariate Analyses of Patient, Antepartum, and Delivery Characteristics Associated with Postpartum Long-Acting Reversible Contraceptive (LARC) Use.

Characteristic	Relative Risk	Confidence Interval	P-Value
Work at agribusiness compared to unemployed	3.5	1.1,11.3	0.032

Note: this model used a Poisson regression with robust error variance; variables different in bivariate comparisons to <0.20 were employment status and parity (bivariate comparisons not shown). Only significant outcomes are presented in above table.

**Table 6:** Multivariate Analyses of Patient, Antepartum, and Delivery Characteristics Associated with Postpartum Sterilization.

Characteristic	Relative Risk	Confidence Interval	P-Value
Parity of greater than 1 compared to 1	3.6	2.5,4.9	<0.001
For every year of increase in age, associated likelihood of sterilization	1.1	1.01,1.08	0.01
Skilled birth attendant compared to unskilled	1.8	1.1,2.9	0.02
Cesarean birth compared to vaginal birth	2.1	1.4,3.1	<0.001

Note: this model used a Poisson regression with robust error variance because the prevalence of sterilization was 18%; variables different in bivariate comparisons to <0.20 were parity, education, age, desired birth attendant, desired birth location, actual birth attendant, mode of birth and actual birth location (bivariate comparisons not shown). Only significant outcomes are presented in above table, with each variable having an independent association with the outcome.

## References

[1] Kantorová V, Wheldon MC, Ueffing P, Estimating progress towards meeting women’s contraceptive needs in 185 countries: A Bayesian hierarchical modelling study. PLOS Medicine 17 (2020): e1003026. 32069289 10.1371/journal.pmed.1003026 PMC7028249

[2] Donovan P, Wulf D. Family planning can reduce high infant mortality levels. Issues in brief (Alan Guttmacher Institute) (2002): 1–4. 12134892

[3] ECONOMIC UNDF, AFFAIRS S. Family Planning and the 2030 Agenda for Sustainable Development (data Booklet). UN (2019).

[4] Post M. HTSP 101: everything you want to know about healthy timing and spacing of pregnancy. Washington, DC: Extending Service Delivery Project (2008).

[5] Nations U. Contraceptive Use by Method 2019: Data Booklet (2021).

[6] Ministerio de Salud P˙blica y Asistencia Social MG, Instituto Nacional de EstadÌstica INEG, SecretarÌa de PlanificaciÛn y ProgramaciÛn del la Presidencia SnG, International ICF. Encuesta nacional de salud materno infantil 2014–2015: informe final (2017).

[7] Asturias EJ, Heinrichs G, Domek G, The center for human development in Guatemala: an innovative model for global population health. Advances in pediatrics 63 (2016): 357–387. 27426907 10.1016/j.yapd.2016.04.001

[8] Pasha O, Goudar SS, Patel A, Postpartum contraceptive use and unmet need for family planning in five low-income countries. Reproductive health 12 (2015): 1–7. 26063346 10.1186/1742-4755-12-S2-S11 PMC4464604

[9] Kestler E, Orozco MdR, Palma S, Initiation of effective postpartum contraceptive use in public hospitals in Guatemala. Revista Panamericana de Salud Pública 29 (2011): 103–107. 21437367 10.1590/s1020-49892011000200005

[10] Curtis KM, Mohllajee AP, Peterson HB. Regret following female sterilization at a young age: a systematic review. Contraception 73 (2006): 205–210. 16413851 10.1016/j.contraception.2005.08.006

[11] Secura GM, Allsworth JE, Madden T, The Contraceptive CHOICE Project: reducing barriers to long-acting reversible contraception. American journal of obstetrics and gynecology 203 (2010): 115.e1–115.e7. 20541171 10.1016/j.ajog.2010.04.017 PMC2910826

[12] Austad K, Shah P, Rohloff P. Correlates of long-acting reversible contraception uptake among rural women in Guatemala. Plos one 13 (2018): e0199536. 29949633 10.1371/journal.pone.0199536 PMC6021094

[13] Waugh LJ. Beliefs associated with Mexican immigrant families’ practice of la cuarentena during postpartum recovery. Journal of Obstetric, Gynecologic & Neonatal Nursing 40 (2011): 732–741. 10.1111/j.1552-6909.2011.01298.x 22092447

[14] Bahamondes L, Villarroel C, Frías Guzmán N, The use of long-acting reversible contraceptives in Latin America and the Caribbean: current landscape and recommend-dations. Human reproduction open 2018 (2018): hox030. 10.1093/hropen/hox030 PMC6276683 30895242

[15] Ponce de Leon RG, Ewerling F, Serruya SJ, Contraceptive Use in 23 Countries in Latin America and the Caribbean: Long-Acting Reversible Contraceptives Much Less Popular than Short-Acting Contraceptives. Global Health 7 (2019): e227–e235. 30683240 10.1016/S2214-109X(18)30481-9 PMC6367565

